# Assessment of Awareness and Preparedness About COVID-19 for Oncology Nurses in Saudi Arabia

**DOI:** 10.7759/cureus.28461

**Published:** 2022-08-27

**Authors:** Amnah F Aluneizi, Dalyal N Alosaimi

**Affiliations:** 1 Oncology, King Salman Armed Forces Hospital, Tabuk, SAU; 2 College of Nursing, King Saud University Riyadh, King Saud University, Riyadh, SAU

**Keywords:** covid-19, awareness, knowledge, preparedness, oncology nursing

## Abstract

Background: As is widely known, preparing nurses to deal with patients infected with COVID-19 is crucial, and knowledge about COVID-19 is needed. However, few researchers have discussed concerns about the overall healthcare system’s preparedness for COVID-19 or oncology nurses’ awareness and preparedness (A&P) regarding the disease, especially in Saudi Arabia.

Objective: Provide a broader understanding of oncology nurses’ A&P regarding the COVID-19 pandemic in Saudi Arabia.

Methods: This was a cross-sectional descriptive study to assess the oncology nurses’ awareness and preparedness regarding the COVID-19 pandemic at King Khaled University Hospital in Riyadh (KKUH) and King Salman Armed Forces Hospital Tabuk (KSAFHT). The population of the study was oncology nurses who were working in day-case, clinic, and inpatient stations in these two hospitals, as well as oncology-certified nurses who were not working in oncology but volunteered to be part of the study. The calculated sample size was 146 nurses. These nurses were selected via convenience sampling. The data were collected with the help of a predesigned questionnaire that was used to assess Libyan hospitals’ preparation for the COVID-19 pandemic and the preparedness of healthcare workers survey. The Statistical Package for Social Sciences Software (SPSS) version 23.0 (SPSS Inc., Chicago, IL) was used to enter and analyze data.

Results: According to the data collected from 146 oncology nurses, as per the study’s knowledge score criteria, 136 participants (93.15%) had a high level of awareness regarding COVID-19, and only 10 participants (6.85%) had a low level of awareness. The nurses’ level of awareness did not show significant association with the age (p = 0.394), gender (p = 0.582), experience (p = 0.566), or education (p = 0.062) of the study participants. Furthermore, the *p-*value showed no statistically significant difference among awareness scores in relation to the designation of nurses. On the other hand, 135 study participants (92.47%) had adequate preparedness and only 11 participants (7.53%) had inadequate preparedness. The level of preparedness was associated with the age, gender, experience, and educational status of study participants with the help of the chi-square test.

Conclusion: The results of this study revealed that the nurses working in the oncology departments in two key Saudi hospitals have sufficient awareness and are prepared to deal with COVID-19 patients. Although nurses working in oncology departments are not primarily responsible for taking care of COVID-19 patients, they may still encounter COVID-19 patients, and they must take all possible protective measures to minimize the spread of COVID-19, as well as provide proper diagnosis and care for these patients.

## Introduction

The practice of oncology nursing covers many areas, such as the pediatric, surgical, medical, gynecology, critical care, and radiation departments. The novel coronavirus disease (COVID-19) presents a greater danger to nurses, especially those who deal with infected patients, than to other medical personnel [1]. Oncology patients generally require special care, especially if they are immunocompromised. Thus, the main concern of oncology nurses with respect to COVID-19 is to prevent and minimize the incidence of COVID-19 infection and its complications [2]. So, healthcare providers should follow strict preventive measures such as using personal protective equipment (PPE) when dealing with or providing care to these patients [3]. One of the main roles of oncology nurses is to train their patients on how to protect themselves by strictly following self-protection techniques in their daily lives [2]. It is widely acknowledged that preparing oncology nurses to deal with patients infected with COVID-19 is crucial, and knowledge about COVID-19 is needed. At the end of 2019, a new coronavirus disease (COVID-19) was found to cause severe pneumonia in the state of Wuhan in China [4].

By early March 2020, the World Health Organization (WHO) had declared the COVID-19 outbreak a worldwide pandemic. By September 2020, more than 30 million people had been infected with the SARS-CoV-2 virus, and more than one million people had died worldwide. In Saudi Arabia, more than 300,000 residents and citizens were reported as active cases with a total of 4,000 deaths, which was in September 2020 [5]. The death rate of infected individuals was higher among the elderly and individuals with chronic and respiratory diseases [6]. Also, population-based studies from China and Italy indicated that the death rate from COVID-19 was higher than reported among cancer patients. However, there is a gap in the available information about how aspects of cancer and its treatment confer a risk of severe COVID-19 [7].

A literature search supported the importance of nurses’ preparedness for crisis times. Few researchers have discussed concerns about the overall healthcare system’s preparedness for COVID-19 and oncology nurses’ awareness and preparedness (A&P) regarding the disease. However, several concerns have been raised regarding specific countries’ preparedness and their ability to control the pandemic [1]. To date, the aggressive battle against COVID-19 is still ongoing, and the WHO is expecting a third wave of the pandemic in Europe [8]. People’s understanding of and compliance with precautionary measures is one of the key factors in defeating this virus. Providing nursing care to oncology patients can be sensitive and require special training because of the nature of the patient's condition. The current study was designed to provide a broader understanding of oncology nurses’ A&P regarding the COVID-19 pandemic in Saudi Arabia.

The aim of this study was to provide a broader understanding of oncology nurses’ A&P regarding the COVID-19 pandemic in Saudi Arabia.

## Materials and methods

This was a cross-sectional study. Data collection was carried out between February 2021 and April 2021. Data for the present study were collected from two settings-one affiliated with the Ministry of Health (MOH) and the other affiliated with the Armed Forces Medical Services. The two settings were King Khaled University Hospital in Riyadh (KKUH) and King Salman Armed Forces Hospital Tabuk (KSAFHT). The population of the study included all oncology nurses who were working in the day case, clinic, and inpatient stations in the two hospitals, as well as oncology-certified nurses who were not working in oncology but volunteered to be part of the study. The sample for the present study included oncology nurses who worked in the day case department, the outpatient clinic, and the inpatient station at KKUH or KSAFHT, as well as oncology-certified nurses who were not working in oncology departments. A convenience sampling strategy was used to recruit participants who were willing to participate. The estimated sample size of 146 nurses was calculated using a 90% confidence level and 6% absolute precision. The expected percentage of preparedness for COVID-19 among nurses was 26.3%. The filled questionnaires and the responses were saved in a database by using passwords and limiting access to the device. Appreciation was expressed for each of the participants. Libyan hospitals’ preparation for the COVID-19 pandemic and the preparedness of healthcare workers survey were used in this study. The survey consisted of three sections. The first section included the participants’ demographic information such as their age, gender, years of work experience in oncology, sources of knowledge, and previous outbreak experience. The second part of the survey included seven items aimed at assessing the awareness and understanding of COVID-19 among nurses. The third part included 11 elements designed to evaluate the nurses’ overall preparedness for managing COVID-19 cases [1-9]. The survey collected data about the information sources regarding COVID-19 that the participants used, their training experience regarding COVID-19, their diagnosis and the management of COVID-19, their use of PPE, their safety protocols, their isolation procedures, their infection control steps, and their reporting procedures [1-9]. Some of the survey questions were yes or no questions, and the others were multiple-choice questions [1,9].

For the awareness assessment section, the possible total scores ranged from zero, which indicated a low level of awareness regarding COVID-19, to seven, which indicated a high level of awareness regarding COVID-19. Each correct answer was given a score of one to reflect the awareness level of the given participant, and a zero was given to each incorrect answer. However, for the preparedness survey, the total score ranged from 0, which indicated insufficient preparedness, to 11, which indicated sufficient preparedness. Those with scores greater than five on the awareness scale were considered to have a high level of awareness, and those who scored greater than eight on the preparedness scale were considered prepared for the crisis [1-9]. The validity of this questionnaire was assessed with the help of intra-class correlation. It was validated in a pilot study of 25 participants who did not participate in the final analysis. An intra-class correlation coefficient was calculated for both the awareness and preparedness scores. For the awareness score, the ICC value was 0.651, which shows moderate reliability. While for the preparedness score, the ICC value was 0.782, which shows excellent reliability.

Data analysis

The Statistical Package for Social Sciences Software (SPSS) version 23.0 was used to analyze and code the data used in this study (SPSS Inc., Chicago, IL). To identify significant findings and a relationship between the study’s variables, descriptive and inferential statistical analyses were used. Descriptive statistics were utilized to describe the study variables. The chi-square test and the Kruskal-Wallis test, a ranked-based nonparametric test, were used to assess for statistically significant differences in the level of awareness and preparedness of the oncology nurses in relation to participants’ characteristics. Missing data were deleted to eliminate missing data, and a new entry for the missing value was updated with another participant’s response. Statistical significance was considered for P < 0.05. To simplify the results, the data was organized in tables and graphs.

## Results

The mean awareness score for the study participants was 6.09 ± 0.92. As per the awareness score criteria, 136 participants (93.15%) had high awareness regarding COVID-19, and only 10 participants (6.85%) had low awareness. The nurses’ level of awareness did not show a significant association with their age (p = 0.394), gender (p = 0.582), experience (p = 0.566), or education (p = 0.062). We assessed the nurses’ awareness scores in relation to their work experience and educational status. The mean awareness scores for nurses with 1-5 years of experience and 5-10 years of experience were 6.09 ± 0.94 and 6.02 ± 0.87, respectively. The mean awareness scores for nurses with 10-15 years of experience and nurses with over 15 years of experience were 6.25 ± 0.89 and 5.96 ± 1.05, respectively. There was no significant difference among the awareness scores across these experience categories (p = 0.516). The mean awareness score was highest for nurses with a diploma (6.20 ± 0.83), followed by nurses with a master’s degree (6.09 ± 1.09). The lowest mean score was seen for nurses with a bachelor’s degree (5.91 ± 0.99) (p-value = 0.315). Among the designations, the highest mean awareness score was for in-charge nurses (6.23 ± 0.83), followed by clinical nurses (6.15 ± 0.86), and nursing managers (5.76 ± 1.16). The p-value showed no statistically significant difference among awareness scores in relation to the designation of nurses (p = 0.692). The mean preparedness score for the study participants was 9.88 ± 1.46. The minimum and maximum scores were 2 and 11. For preparedness, the criteria were set as adequate and inadequate. Participants with scores of over 8 were labeled as having adequate preparedness, and those whose scores were less than 8 were labeled as having inadequate preparedness. As per the aforementioned criterion, 135 study participants (92.47%) had adequate preparedness and only 11 participants (7.53%) had inadequate preparedness. The level of practice was associated with the study participants' age, gender, experience, and educational status. All variables had no significant impact on the level of preparedness (i.e., age, p-value = 0.278; gender, p-value = 0.180; experience, p-value = 0.602; and education, p-value = 0.808). We assessed the preparedness scores in relation to the nurses’ experience, educational status, and designation. The p-value showed no significant difference in knowledge scores across these experience categories (p-value = 0.187). The mean preparedness score was highest for nurses having a diploma (9.94 ± 1.36) followed by nurses with a bachelor’s degree (9.87 ± 1.30) and nurses with a master’s degree (9.67 ± 2.10) (p-value = 0.930). The highest preparedness score was for clinical nurses (10.04 ± 1.36) followed by nursing managers (9.56 ± 1.781) and in-charge nurses (9.48 ± 1.470). Significant differences were seen among the mean preparedness scores for different designations of nurses. The multiple comparison test showed no significant difference in preparedness scores between in-charge nurses and nurse managers (p-value = 0.927). Significant differences were seen in the preparedness scores between in-charge nurses and clinical nurses (p-value = 0.046) and nursing managers and clinical nurses (p-value = 0.043) (Tables 1-4).

**Table 1:** Demographic characteristics of the nurses

	Frequency	Percentage
Age		
20–29 years	33	22.6%
30–39 years	93	63.7%
40+ years	20	13.7%
Gender		
Male	4	2.7%
Female	142	97.3%
Marital status		
Single	62	42.5%
Married	84	57.5%
Experience		
1–5 years	33	22.6%
5–10 years	44	30.1%
10–15 years	44	30.1%
15+ years	25	17.1%
Educational status		
Bachelor’s degree	80	54.8%
Diploma	45	30.8%
Master’s degree	21	14.4%
Designation		
Clinical nurse	100	68.5%
In-charge nurse	21	14.4%
Nurse manager	25	17.1%
Working department		
Certified oncology nurses not working in oncology	44	30.1%
Oncology outpatient unit	27	18.5%
Oncology day case unit	36	24.7%
Oncology inpatient	39	26.7%
Work directly with suspected COVID-19 cases		
Yes	62	42.5%
No	84	57.5%
Source of information about the COVID-19 outbreak		
Training courses	67	45.9%
Medical leaflets	51	34.9%
Media	38	26%
Social networks	85	58.2%
Official sources, WHO, MOH	122	83.6%

**Table 2:** Percentage of correct answers for awareness and preparedness of COVID-19

Sn	Awareness (N = 146)		Answered correctly	
			n	%
1	What are the symptoms of COVID-19 infections?		146	100%
2	How to diagnose COVID-19?		93	63.7%
3	COVID-19 case definition		136	93.2%
4	Identification of at-risk patients		135	92.5%
5	How to prevent transmission of COVID-19?		129	88.4%
6	Knowledge of personal protective equipment		145	99.3%
7	Alcohol-based hand rub is sufficient to kill SARS-CoV-2		107	73.3%
Mean awareness score = 6.09±0.92, min=3 and max=7				
Level of awareness		<5	10	7%
		>5	136	93%
Mean preparedness score = 9.88±1.46, min=2 and max-11				
Level of preparedness		Adequate > 8	135	92.47%
		Inadequate < 8	11	7.53%

**Table 3:** Level of COVID-19 awareness and preparedness in relation to participants’ characteristics

	Level of awareness				p-value*	Level of preparedness				p-value*
	Low		High			Adequate		Inadequate		
	n	%	n	%		n	%	n	%	
Age										
20–29 Years	4	40%	29	21.3%	0.394	32	23.7%	1	9.1%	0.278
30–39 Years	5	50%	88	64.7%		86	63.7%	7	63.6%	
40+ Years	1	10%	19	14%		17	12.6%	3	27.3%	
Gender										
Male	0	0%	4	2.9	0.582	3	2.2%	1	9.1%	0.180
Female	10	100%	132	97.1%		132	97.8%	10	90.9%	
Experience										
1–5 Years	3	30%	30	22.1%	0.566	32	23.7%	1	9.1%	0.602
5–10 Years	2	20%	42	30.9%		41	30.4%	3	27.3%	
10–15 Years	2	20%	42	30.9%		40	29.6%	4	36.4%	
15+ Years	3	30%	22	16.2%		22	16.3%	3	27.3%	
Education										
Bachelor’s degree	2	20%	78	57.4%	0.062	75	55.6%	5	45.5%	0.808
Diploma	6	60%	39	28.7%		41	30.4%	4	36.4%	
Master’s degree	2	20%	19	14%		19	14.1%	2	18.2%	

**Table 4:** Association of awareness and preparedness score in relation to different factors

Categories	Awareness score			p-value*	Preparedness score			p-value*
	n	Mean	SD		n	Mean	SD	
Clinical experience								
1–5 years	33	6.09	0.94	0.516	33	10.18	1.50	0.187
5–10 years	44	6.02	0.87		44	9.86	1.30	
10–15 years	44	6.25	0.89		44	9.73	1.66	
15+ years	25	5.96	1.05		25	9.76	1.33	
Education								
Bachelor’s degree	45	5.91	0.99	0.315	45	9.87	1.30	0.930
Diploma	80	6.20	0.83		80	9.94	1.36	
Master’s degree	21	6.09	1.09		21	9.67	2.10	
Designation								
Clinical nurse	100	6.15	0.86	0.692	100	10.04	1.363	0.033
In-charge nurse	21	6.23	0.83		21	9.48	1.470	
Nurse managers	25	5.76	1.16		25	9.56	1.781	

## Discussion

In this study, we assessed the knowledge and preparedness regarding COVID-19 of nurses working in oncology departments in Saudi Arabia. We included 146 oncology nurses. Among these nurses, 62 (42.5%) were directly working with COVID-19 patients, and 84 (45.9%) were not directly working with COVID-19 patients. The mean awareness score for study participants was 6.09 ± 0.92. As per the scoring criteria, 93.15% of the study participants had high awareness, and only 10 (6.85%) had low awareness.

The research by Shawahna and Jaber [9] evaluated nurses’ knowledge, attitudes, and application of preventive measures against COVID-19. They found that female nurses had superior knowledge to male nurses, and only 30.8% of nurses achieved 80% or more correct answers on a knowledge exam [10]. However, in the present study, the awareness scores of nurses were higher than those in the study by Shawahna and Jaber [9]. In this study, 93.15% of nurses had adequate knowledge regarding COVID-19. This might be due to the fact that among these nurses, 83.67% were receiving COVID-19 information from official sources (the WHO and MOH). In our study, there were far fewer male nurses than female nurses. So, no such association was observed in this study. However, other studies have reported significant gendered differences in nurses’ knowledge [9].

Nemati et al. [10] assessed nurses’ degree of awareness. Over half of the nurses studied (56.5%) had a near-perfect understanding of COVID-19-related material (e.g., sources, transmission, signs and symptoms, prognosis, treatment, and mortality rate). Their total knowledge scores, on the other hand, did not vary substantially by age, education level, or job experience. The researchers hypothesized that more knowledge might result in improved control of infectious illnesses such as COVID-19 [10]. Numerous studies from around the world have shown that healthcare professionals are generally knowledgeable about COVID-19 [11-13]. According to the research by Desalegn et al. [12], 58.7% of healthcare professionals had moderate knowledge, 26.5% had excellent knowledge, and 14.7% had low knowledge regarding COVID-19. Younger age groups, females, and non-physicians all had lower knowledge ratings [12]. Prior research has established that one’s degree of knowledge about a certain illness may affect one’s attitudes and that erroneous behavior and attitudes directly increase one’s chance of infection. In general, a lack of knowledge and the presence of misconceptions among healthcare workers result in diagnostic delays, increased disease transmission, and ineffective infection control measures. Numerous studies conducted in a variety of contexts have shown significant disparities in the knowledge, awareness, attitudes, practice, and readiness of healthcare workers in the battle against the pandemic [14].

Globally, significant variation has been observed in health professionals’ awareness of and readiness for COVID-19. However, knowledge may have a variety of effects on an individual’s attitudes, behaviors, and positive attitudes and, therefore, may alter behavior in a wider context [15]. Elhadi et al. [1] assessed doctors’ and nurses’ knowledge and readiness regarding COVID-19. According to their findings, just 26.5% of participants had sufficient knowledge and awareness about COVID-19, and 73.5% possessed only a rudimentary understanding. Only 7.8% were well prepared to cope with COVID-19 outbreaks, while the remaining 92.2% had a readiness score of 8, indicating insufficient preparedness. This research was performed in a resource-constrained environment.

In research conducted in Jazan, Saudi Arabia, most respondents were found to be aware of the information and preventative measures about COVID-19, and they were well-equipped to combat COVID-19 [15]. Healthcare professionals were included in the study’s population (36.7% of the respondents were nurses, physicians, or pharmacists). This research also demonstrated that knowledge and preparation do transfer into better COVID-19 preventive measures [15]. Turkish research on oncology nurses’ understanding of COVID-19 found that oncology nurses’ experiences with COVID-19 accounted for 29.1% of their knowledge level regarding COVID-19 [15]. Nurses’ education levels, their having a relative diagnosed with COVID-19, and their compliance with COVID-19 standards have also been shown to have a statistically significant effect on their COVID-19 knowledge levels [14]. In research conducted in Saudi Arabia, the educational level of nurses was shown to influence their understanding of COVID-19 [16]. According to Zhang et al. [17], healthcare professionals’ education level influenced their knowledge, and those with a postgraduate degree had greater knowledge regarding COVID-19 [18].

In this study, no significant difference was seen in knowledge scores among nurses with different educational backgrounds. However, nurses with master’s degrees and diplomas had higher knowledge scores than nurses whose highest degree was a bachelor’s degree. It was also observed that among nurses with a higher level of awareness, the highest proportion had a bachelor’s degree, followed by nurses with a diploma. Only 14% of the nurses with higher awareness had a master’s degree. In this study, about 54.8% of the nurses surveyed had a bachelor’s degree, and 14.4% had a master’s degree.

The results regarding preparedness showed that in this study, 92.47% of the oncology nurses had adequate preparedness for COVID-19, and only 7.53% were inadequately prepared for COVID-19. Nurses’ characteristics such as age, gender, education, and experience were not significantly associated with their preparedness level. Nurses from all age groups, both genders, and with all levels of educational background and experience were adequately prepared for dealing with COVID-19 infection. The preparedness domain consisted of 11 questions, and for 10 questions, more than 80% of the nurses’ responses were correct. Still, overall, the responses regarding preparedness were high, thus indicating the oncology nurses’ strength and working capacity to deal with the COVID-19 pandemic.

The present study indicated that the oncology nurses were aware of social isolation, proper hand hygiene, the use of face masks, and the avoidance of travel. These are the desired actions that must be carefully adhered to in order to halt the progress of the illness. Tripathi and her Jazan team [15] found that all healthcare professionals in Jazan hospital expressed readiness to cope with COVID-19. Nurses must be knowledgeable about COVID-19 to perform their duties and responsibilities in these five areas [2].

Additionally, it is critical that oncology nurses use current information, such as information about COVID-19 testing, self-isolation, social distancing, quarantine, and therapy, as well as personal protective equipment, to safeguard oncology patients from COVID-19 infection [3]. The role and contribution of nurses are more critical than ever during this epidemic. COVID-19 creates substantial barriers to providing high-quality treatment to oncology patients. Thus, nursing administration and hospital administration should have a structured training program in place to avoid hospital infections during this unusual epidemic.

Proper information combined with a positive attitude has been shown to result in the appropriate deployment of protective measures at work, and as a result, the chance of contracting illness may be reduced [2,3,19]. Nurses’ commitment to deploying preventive measures against COVID-19 is very clearly influenced by their knowledge and attitudes toward the illness. Thus, evaluating nurses’ knowledge, attitudes, and implementation of preventive measures against COVID-19 is critical. Additionally, nurses serve on the front lines, giving direct treatment to COVID-19 patients. Additional work is required to create strategic suggestions and incorporate newly acquired information into nurses’ schooling. Efforts to contain and prevent COVID-19, as well as to care for individuals who have been affected, are ongoing [3].

## Conclusions

The results of the current study demonstrated that the oncology nurses had sufficient awareness and preparedness regarding COVID-19 infection in Saudi Arabia. Characteristics like age, gender, educational status, and experience had no significant impact on the awareness and preparedness of oncology nurses. This suggests that the Saudi government is implementing and making use of all available resources to enable Saudi healthcare workers to combat COVID-19.
